# Biomaterial-Based Activation and Expansion of Tumor-Specific T Cells

**DOI:** 10.3389/fimmu.2019.00931

**Published:** 2019-05-03

**Authors:** Marjolein Schluck, Roel Hammink, Carl G. Figdor, Martijn Verdoes, Jorieke Weiden

**Affiliations:** ^1^Department of Tumor Immunology, Radboud Institute for Molecular Life Sciences, Radboud University Medical Center, Nijmegen, Netherlands; ^2^Division of Immunotherapy, Oncode Institute, Radboud University Medical Center, Nijmegen, Netherlands; ^3^Institute for Chemical Immunology, Nijmegen, Netherlands

**Keywords:** cancer immunotherapy, biomaterials, T cells, artificial antigen-presenting cells, scaffold, anti-tumor immune response, synthetic immune niche, molecular cues

## Abstract

Traditional tumor vaccination approaches mostly focus on activating dendritic cells (DCs) by providing them with a source of tumor antigens and/or adjuvants, which in turn activate tumor-reactive T cells. Novel biomaterial-based cancer immunotherapeutic strategies focus on directly activating and stimulating T cells through molecular cues presented on synthetic constructs with the aim of improving T cell survival, more precisely steer T cell activation and direct T cell differentiation. Synthetic artificial antigen presenting cells (aAPCs) decorated with T cell-activating ligands are being developed to induce robust tumor-specific T cell responses, essentially bypassing DCs. In this perspective, we approach these promising new technologies from an immunological angle, first by identifying the CD4^+^ and CD8^+^ T cell subtypes that are imperative for robust anti-cancer immunity and subsequently discussing the molecular cues needed to induce these cells types. We will elaborate on how biomaterials can be applied to stimulate T cells *in vitro* and *in vivo* to improve their survival, activation and function. Scaffold-based methods can also be used as delivery vehicles for adoptive transfer of T cells, including tumor-infiltrating lymphocytes (TILs) and chimeric antigen receptor expressing (CAR) T cells, while simultaneously stimulating these cells. Finally, we provide suggestions on how these insights could advance the field of biomaterial-based activation and expansion of tumor-specific T cells in the future.

## Introduction

Immunotherapy provides a revolutionary treatment modality for cancer. A variety of strategies have been developed to improve the clinical outcome of patients by generating long-term anti-tumor immune responses. The development of therapeutic monoclonal antibodies that block co-inhibitory receptors on T cells, such as cytotoxic T-lymphocyte-associated antigen 4 (CTLA-4) and programmed death 1 (PD-1), has shown exceptional clinical benefit in cancer patients and is seen as a crucial breakthrough in the cancer immunotherapy field (1).

Apart from relieving suppression in pre-existing T cells, other immunotherapeutic strategies focus on increasing the number of tumor-reactive T lymphocytes that recognize either tumor-specific antigens, tumor-associated antigens, cancer-testis antigens or neo-antigens. Dendritic cell (DC) vaccination targets antigen-presenting DCs, which are capable of priming T cells by capturing, processing and presenting antigens to naïve T cells together with co-stimulatory cues (2). To generate DC vaccines, patient-derived DCs are cultured *ex vivo*, maturated and loaded with antigens, after which they are infused back into the patient where they activate tumor-reactive T cells (3). Increased overall survival, functional tumor-specific immune responses and low toxicities have been observed with this strategy (4, 5). Other cell-based therapies attempt to increase the number of circulating tumor-specific T cells by reinfusing autologous *ex vivo*-expanded T cells derived from tumors (tumor-infiltrating lymphocytes, TIL) or genetically engineering them to confer tumor reactivity using high affinity T-cell receptors (TCR) or chimeric antigen receptors (CAR) (6). Promising preclinical data have been obtained with these adoptive T-cell therapies (1, 7, 8). The prominent immunotherapeutic strategies described above all focus on generating robust tumor-directed T cell responses, which is crucial to inducing effective and long-lasting anti-tumor immunity, as there is a strong correlation between tumor-infiltrating CD8^+^ T cells and patient survival in virtually all cancer types (9). In addition, antigen-specific CD4^+^ T helper cells are believed to be critically involved in the induction of optimal anti-tumor responses (10, 11). It is therefore evident that T cells play a central role in cancer immunotherapy.

Although current cancer immune therapies have shown promising preclinical and clinical results, challenges remain that may limit therapeutic benefit. To improve on this and to design new therapeutic strategies, interest in the field of biomaterial engineering has grown. Biomaterials have proven valuable in reducing systemic toxicities, enhancing accumulation in tumors, improving pharmacokinetics and ensuring sustained release by controlled (targeted) drug delivery (12, 13). Biomaterial-based immunotherapeutic strategies led to the development of nanoparticles for the targeted delivery of cargo to immune cells *in vivo*, such as cytokines, DC-activating agents or small inhibitors (13–15). Careful design can be applied to tune the delivery of DC-targeted vaccines using materials responsive to temperature (16) or pH (17, 18). Other biomaterial-based approaches focus on improving *ex vivo* immune cell expansion or on supporting immune cells after adoptive transfer (13, 14). Furthermore, there has been a rise in the development of synthetic, acellular artificial antigen presenting cells (aAPCs) that can target and activate T cells directly (19, 20), thereby bypassing the need for DC activation. By presenting molecular cues on synthetic constructs based on biomaterials, specific signals are transmitted to T cells in a well-defined context and controlled manner to support T cell viability, activation and differentiation.

In this perspective, we will detail what T cell subtypes are imperative for robust anti-cancer immunity and which molecular cues are needed to induce these T cells. Next, we will elaborate on how these molecular cues can be presented by biomaterials for direct activation and expansion of T cells. The use of biomaterials to aid the adoptive transfer of T cells will also be discussed. Finally, we will illustrate in which direction the field of biomaterial engineering for cancer immunotherapy should go for the next generation of biomaterial-based cancer immunotherapies.

## T Cell Subsets in Cancer Immunotherapy

**Figure 1:**
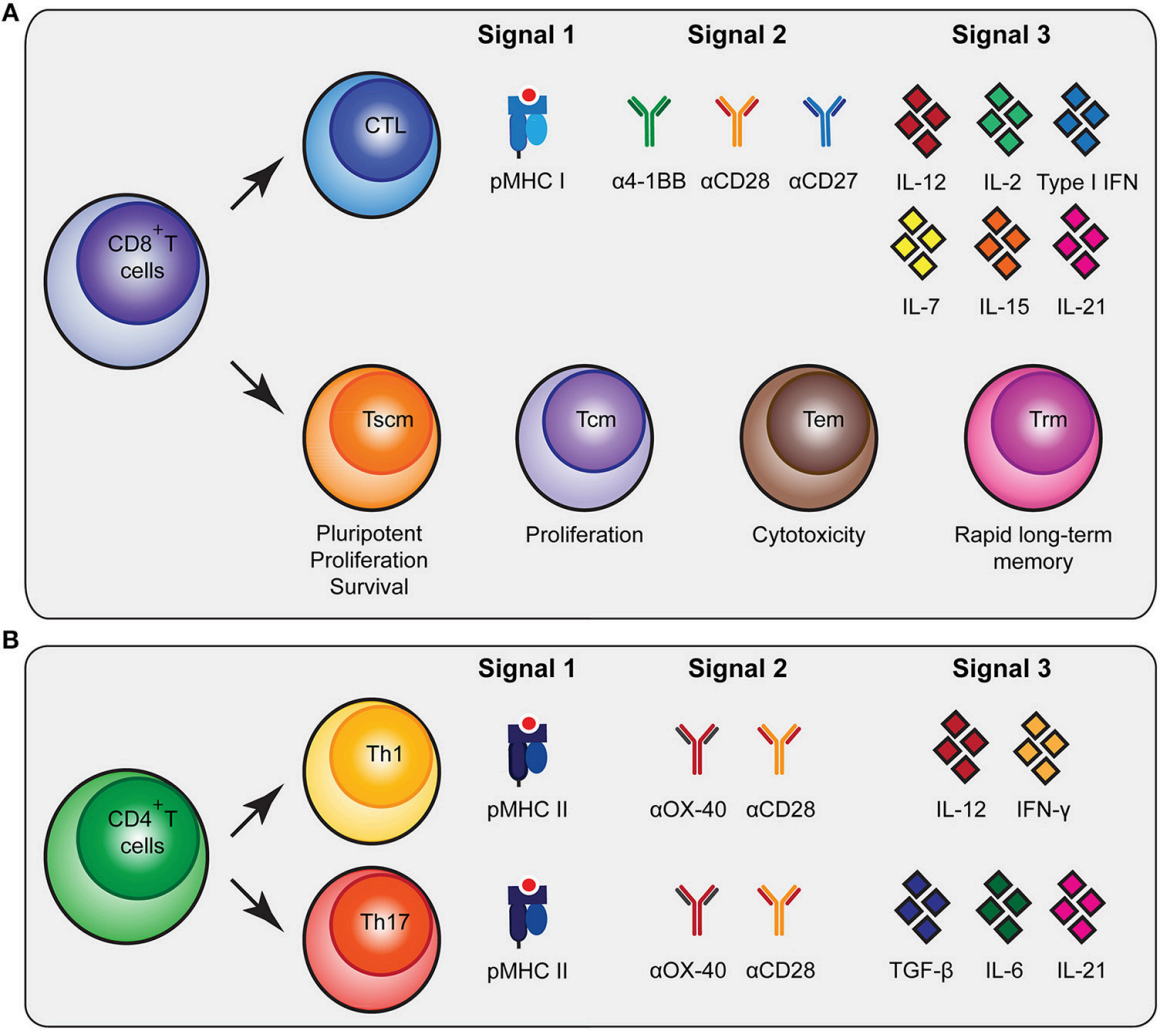
Molecular cues involved in CD8^+^ and CD4^+^ T cell activation and differentiation. **(A)** CD8^+^ T cells can be subdivided in cytoxic T lymphocytes (CTLs) and memory subsets [memory stem cells (Tscm), central memory (Tcm), effector memory (Tem) and tissue-resident memory (Trm)] that all have specific functionalities. To stimulate antigen-specific CTLs, biomaterials should present peptide MHC (pMHC) class I, agonistic antibodies that trigger co-stimulatory receptors for signal 2 and cytokines as signal 3 as depicted. **(B)** To trigger differentiation of CD4^+^ T cells into T helper 1 (Th1) and Th17 cells, biomaterials need to present pMHC class II together with co-stimulatory signals and different combinations of cytokines. As an alternative to agonistic antibodies to trigger co-stimulatory signaling pathways, natural ligands of co-stimulatory receptors can be used.

To generate durable anti-tumor immune responses that have a beneficial impact on the clinical outcome of cancer patients, potent CD8^+^ and CD4^+^ T cell responses are crucial (9–11). Here, we will discuss the roles of different T cell subtypes in cancer-specific immune responses and we will highlight the cellular and molecular characteristics of these T cells (Figure 1).

Upon interaction with their cognate antigen in the context of major histocompatibility complex class I (MHC I) and co-stimulatory cues, CD8^+^ T cells will undergo extensive proliferative expansion to create a large population of short-lived effector cytotoxic T lymphocytes (CTLs) that have tumor-killing capacities. The CTL population comprises functionally distinct subsets (21). For instance, expression of CX3CR1 on CTLs is associated with their ability to generate memory subsets and serves as a predictor for CX3CR1 expression on the generated memory cells, which is associated with robust cytotoxic effector functions (22, 23). CXCR5-expressing CTLs are involved in chronic viral infections and show reduced susceptibility to exhaustion (24). Additional heterogeneity may exist regarding cytokine production and the (co-)expression of perforin and various granzymes (25). In addition to these short-lived CTLs, the formation of CD8^+^ memory T cells is required to support long-term anti-tumor immunity. Following a progressive differentiation model, primed naive CD8^+^ T cells (Tn) will progress into different memory T cell populations [T stem cell memory (Tscm), T central memory (Tcm), T effector memory (Tem)] (21, 22, 25–27). The Tscm subset displays increased anti-tumor activity, enhanced proliferation, increased survival capacities and multipotency (27, 28). The Tcm generally have higher proliferative abilities while Tem are more cytotoxic (22). In contrast to circulating memory T cells, there is also a population of non-circulating memory T cells, tissue resident memory T cells (Trm). These Trm cells were shown to be superior in providing rapid long-term protection against recurrent infections (29). Inducing a broad repertoire of potent CTLs together with CD8^+^ memory T cells will be highly beneficial for robust anti-tumor immunity (Figure 1A).

CD4^+^ T cell help is imperative for potent CD8^+^ T cell activation by supplying cytokines and co-stimulation, by enhancing persistence and migration, and by reactivating memory CD8^+^ T cells (30–32). Recently, it has been reported that CD4^+^ T cells are also dependent on CD8^+^ T cells, underlining the mutual dependence of CD4^+^ and CD8^+^ T cell responses (10). Furthermore, a RNA vaccination study clearly showed the importance of CD4^+^ T cell neo-epitopes in controlling murine tumors (11). The CD4^+^ T cell population can be subdivided into specific subsets, each having their own signature cytokine repertoire (33, 34). The T helper 1 (Th1) subset is strongly associated with better prognosis, improved survival, low incidence of tumor recurrence and prolonged disease-free survival in cancer immunology (35). This is in part due to their supportive role in cellular immunity, the cytokines they produce [including interferon-γ (IFN-γ)] (35), and their role in inducing immunological memory (36). In addition, notable results of a mouse melanoma study showed that the T helper 17 (Th17) subset producing IL-17 was involved in B16 tumor rejection (37). Other studies have also indicated a positive role for Th17 cells in the development of long-term anti-tumor immunity and their help in CTL activation and recruitment to the tumor (38, 39). Besides providing support to CTLs, CD4^+^ T cells can also contribute to the anti-tumor immune response independent of CD8^+^ T cells (30, 40, 41) by acquiring cytotoxic activity and executing a direct anti-tumor effect (36, 40). Finally, CD4^+^ T regulatory cells (Tregs) mainly encompass the immune inhibitory subset, which is important in physiological settings to prevent autoimmunity (35). Due to their immune inhibitory profile, Tregs can prevent tumor clearance by inhibiting CTL functions (42).

The generation of the various CD4^+^ T cell subsets *in vitro* is mainly determined by the cytokine profile present during T cell receptor-mediated activation (34). The presence of interleukin-12 (IL-12) and IFN-γ will skew CD4^+^ T cells toward a Th1 profile, while the presence of TGF-β, IL-6 and IL-21 will drive the differentiation toward a Th17 profile (34) (Figure 1B). Besides inducing Th1 differentiation, IL-12 also enhances the proliferation of activated T cells and induces cell-mediated immunity (43). In addition, IL-1 is able to directly act on CD4^+^ T cells, especially IL-17 producing cells, increasing antigen-specific T cell expansion and enhancing survival (Figure 1B) (44).

Taken together, these studies emphasize the importance of inducing potent CTL and stimulatory CD4^+^ T helper cell subsets (in particular Th1 and Th17 cells) to induce potent anti-tumor responses, but they also highlight the need for differentiation of the effector subsets into memory cells to help prevent relapse. Insight into the molecular cues that can optimize the design of biomaterial strategies to gain control over the repertoire of T cells that is induced is therefore pivotal.

## Molecular Cues to Activate and Expand T Cells

To provide T cells with the signals that are required for activation and differentiation, inspiration could be sought in the mechanism of action of natural antigen-presenting cells (APCs) for T cell priming, thereby creating aAPCs. Three fundamental signals for T cell activation are (i) triggering TCR signaling; (ii) adequate co-stimulation, e.g., through the CD28 signaling axis; and (iii) the availability of cytokines to direct T cell differentiation (45, 46). TCR engagement can be mimicked by agonistic αCD3 antibodies for polyclonal T cell expansion or recombinant MHC peptide complexes (pMHC) for antigen-specific T cell expansion. Artificial co-stimulation can be provided using agonistic αCD28 antibodies or recombinant natural ligands CD80 (B7.1) and CD86 (B7.2). Recombinant cytokines that provide signal 3 are widely available and are typically presented in soluble form.

**Figure 2:**
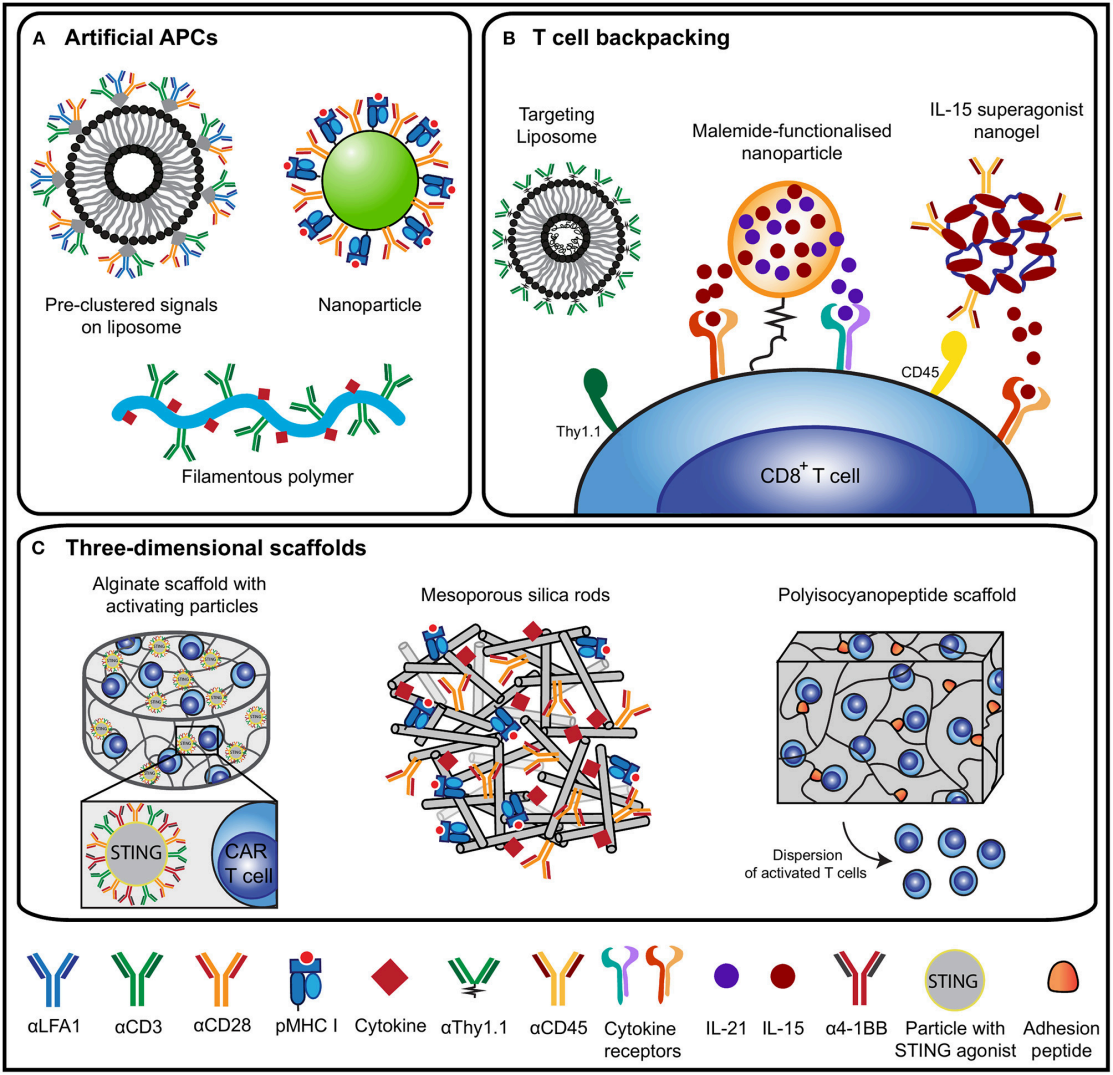
Overview of biomaterial design strategies for T cell activation and expansion. **(A)** An overview of design strategies of artificial antigen-presenting cells (aAPCs) based on liposomes (48), nanoparticles (19) or filamentous polymers (49). aAPC designs present various molecular cues to induce T cell activation, including pMHC or αCD3 antibodies as signal 1, αCD28 antibodies to mimic signal 2 and cytokines as signal 3. **(B)** Different T cell backpacking strategies for the *ex vivo* or *in vivo* targeting of cytokine-loaded particles to T cells using antibody as targets [liposome (50) and nanogel (51)] or through chemical [binding (52) (nanoparticle)]. These strategies ensure targeted delivery of cytokines to support persistence of adoptively transferred cells *in vivo*. **(C)** 3D scaffold-based strategies to expand T cells and to support adoptively transferred (CAR) T cells. Designs include alginate scaffolds with stimulatory microparticles for CAR T cell expansion (53), mesoporous silica rods for T cell activation (54) and a synthetic polyisocyanopeptide-based scaffold that disperses T cells (55).

Various biomaterial designs have been synthesized to mimic DCs which vary in their shape, the signals they present and the method of administration (19, 47) (Figure 2A). Traditionally, soluble polymers or polymeric beads presenting agonistic αCD3/αCD28 antibodies to T cells are used to induce vigorous polyclonal expansion. Besides CD28, co-stimulatory signals belonging to the tumor necrosis factor receptor superfamily (TNFRSF), such as OX-40, 4-1BB, CD27, and LIGHT, are also potentially interesting to steer T cell activation (56, 57). Though OX-40, 4-1BB and CD27 can perform co-stimulation for both CD4^+^ and CD8^+^ T cells, OX-40 was shown to predominantly act as a co-stimulatory molecule for CD4^+^ T cells (58–61). Engagement of 4-1BB and CD27 were more prone to induce potent CD8^+^ activation. An aAPC design presenting αCD3 antibodies with α4-1BB antibodies as the co-stimulatory cue was reported to preferentially expand memory cells and induce enhanced cytolytic activity compared to aAPCs presenting αCD3 and αCD28 (62). CD27 co-stimulation enhances activation and survival of CD8^+^ T cells (60, 61), prevents activation-induced cell death (60) and supports the presence of tumor-specific CD8^+^ T cells residing within established melanoma (63). Expression of LIGHT in the tumor microenvironment of patients increases T cell expansion, activation and infiltration and correlates with improved clinical outcome (64, 65). Furthermore, LIGHT signaling enhanced T cell proliferation, IFN-γ production, tumor infiltration and regression of established tumors in a P815 mastocytoma tumor model and a CT26 colon cancer model (57, 64). These studies imply that careful tuning of co-stimulatory cues presented by biomaterials can steer T cell priming and functionality.

The third signal consisting of cytokines is especially important for naïve CD8^+^ T cells to differentiate, develop their effector functions, and form potent memory populations (66). Absence of this third signal can result in deletion or anergy of the activated cells (67, 68). In a normal immunological setting, CD4^+^ T helper cells promote IL-12 or type I IFN production by DCs in a CD40-dependent manner to ensure potent CTL development (67, 68). Apart from supplying T cells with recombinant cytokines presented by biomaterials, the adaptor molecule Stimulator of IFN Gene (STING) could be used to induce type I IFN production (69, 70). Besides IL-12 and type I IFN, there are various other cytokines involved in T cell activation and differentiation such as IL-2, IL-7, IL-15 and IL-21 (Figure 1A) (66). IL-7, IL-15 and IL-21 are important for CD8^+^ T cell memory formation and maintenance (71), while IL-2 promotes the expansion of both CD4^+^ and CD8^+^ T cells, thus augmenting the effector T-cell response (72). Moreover, cytokines steer CD4^+^ T cell development into the different subsets (34), which emphasizes the necessity to include these signals into a biomaterial design.

## Molecular Cues to Generate T Cells of High Quality

Not only the quantity of the generated T cells is important, as the quality needs also to be considered. When T cells reach a more differentiated state, the cell effector functions increase while the memory functions and proliferation capacity decrease (26). Experimental studies in mice and patients have shown a superior role for less differentiated cells (Tscm and Tcm) in adoptive cell transfer, as was demonstrated by enhanced engraftment, expansion, persistence and anti-tumor responses of these minimally differentiated T cells *in vivo* (28, 73–77).

The low numbers of circulating Tscm (28) cells have resulted in the development of *in vitro* culture practices where the differentiation of naïve T cells is controlled by supplementing culture media with IL-7 plus IL-15 or IL-21 and/or small molecules to activate the Wnt/β-catenin pathway (71, 78). The cells generated with these culture protocols showed increased engraftment, expansion and higher tumor reactivity (71, 78). In addition, longer expansion time of T cells *ex vivo* can also drive T cell differentiation and negatively affect cytolytic activity, proliferation, tumor control and T cell persistence *in vivo* (79).

T cell differentiation may also be influenced by T cell metabolism. TILs cultured with a small inhibitory drug for protein kinase B (AKT) (80), naïve T cells cultured with an inhibitor for mammalian target of rapamycin (mTOR) (81) and CD8^+^ T cells exposed to 2-hydroxyglutarate (82) induces T cells with transcriptional and metabolic properties characteristic of memory T cells that show increased persistence and anti-tumor response *in vivo* after adoptive transfer. These studies indicate the importance of T cell quality and how this may affect persistence, proliferation, survival and effector functions *in vivo*, and demonstrate possibilities for improving *ex vivo* T cell cultures that could also be highly relevant in designing biomaterial-based systems for T cell expansion.

The multifunctionality of the effector T cells also influences the quality of the generated T cells (72). T cells are considered to be multifunctional when having two or more functions including, but not limited to, the production of cytokines, chemokines and/or degranulation (72). Multifunctional CD4^+^ and CD8^+^ T cells are able to secrete more IFNγ, and T cells producing both IFNγ and TNF can mediate more efficient killing compared to single cytokine-producing cells (72). Even though these are illustrations from the field of infectious diseases, multifunctional effector T cell responses could also benefit anti-tumor immunity.

## Designing Biomaterials for Optimal Tumor-Specific T Cell Priming

Biomaterials can be used to present the three imperative signals to T cells to support activation, expansion and differentiation in a spatiotemporally well-defined and sustained manner (83). The defined structural nature of biomaterials enables chemical modification to introduce desired functionalities through standard conjugation or bio-orthogonal chemistries such as “click chemistry” (84, 85). The characteristics of the biomaterial, such as biodegradability, biocompatibility, half-life and the implementation of biological targeting moieties, but also physical properties such as shape, surface topology and mechanical properties, can shape the interaction with T cells and thus alter the immune response that is provoked (86).

The context in which molecular cues are provided can also influence T cell responses. Immobilizing signal 1 on a surface was reported to promote robust T cell activation (87), because physical forces play an important role in TCR signal transduction (88). The relevance of force upon the TCR was interrogated in more detail by making use of materials with different stiffness (89, 90). MHC class I molecules presented on a softer poly(dimethylsioxane) surface led to enhanced T cell proliferation, improved IL-2 production and increased Th1 differentiation compared to TCR engagement using rigid polystyrene beads (89, 90). In addition to the effect of material stiffness, polymeric aAPC presenting T cell-stimulating cues in a multivalent context demonstrated the importance of multivalency for long-lasting T cell activation (91, 92). Furthermore, superior CD8^+^ T cell activation was observed for biomaterials displaying signals 1 and 2 in a pre-clustered manner (19, 47, 93, 94), for instance on liposomes (48). Signal density also affects T cell responses, as CD4^+^ T cells were reported to remain unresponsive when signal 1 is presented in a density that is too low (95–97). In addition to signal density, the quantity of signals 1 and 2 can also influence the effector Th1 CD4^+^ and effector CD8^+^ T cell responses (72). Limited amounts of signal 1 or 2 were shown to induce Th1 cells and CD8^+^ T cells that only secrete IFNγ, while in the presence of increased concentrations of signal 1 or 2 cells both IFNγ and IL-2 were secreted (98, 99).

Most biomaterial designs implement cytokines (signal 3) either in release vesicles or via adsorption (47, 54, 100), creating a local high concentration of soluble cytokines. Robust T cell activation can also be obtained with immobilized cytokines and might preferentially deliver these to T cells through co-presentation of T cell-specific antibodies (49), providing a new range of possibilities for the addition of cytokines in biomaterial-based immune therapies.

When using biomaterials, care must be taken to prevent the induction of exhausted T cells due to persisting T cell stimulation, leading to diminished cytokine production, reduced proliferative capacity, decreased killing abilities, and high expression of co-inhibitory molecules, such as PD-1 and CLTA-4 (101). Therefore, one should not only focus on trying to induce a strong activation signal, but special care should be taken to achieve appropriate stimulation levels to ensure desired T cell activation and prevent T cell exhaustion. To prevent or counteract the exhausted state of T cells, the biomaterial might need to be equipped with PD-1 and CTLA-4 blocking antibodies. Several biomaterial designs have been tested to improve the delivery and sustained release of these antibodies, displaying improved anti-tumor efficacy (14, 102). An alternative strategy to prevent T cell exhaustion could be to provide co-stimulation with an αCD2 antibody (103).

## Biomaterials for Adoptive T Cell Transfer

Apart from applying biomaterials to prime and expand T cells with stimulatory cues, biomaterials are also excellent tools to support the adoptive transfer of T cells for cancer immunotherapeutic purposes (Figures 2B,C). Adoptive T cell therapy (ACT) has shown promising results in inducing durable anti-tumor immune responses (1, 7, 8). However, efficacy generally depends on the co-administration of lymphodepleting chemotherapy and/or high doses of IL-2 to support the persistence of these cells *in vivo* (1, 104, 105). Biomaterials have been designed to reduce toxicities seen with the systemic administration of these adjuvants and to enhance the response of adoptively transferred cells (51, 52) (Figure 2B). In an elegant design of liposome-like synthetic nanoparticles that encapsulate IL-15 superagonist and IL-21, reduced thiol groups on the T cell surface were used to covalently bind nanoparticles onto the cells before ACT (52). These “backpacks” resulted in considerably more proliferation and persistence of the transferred cells *in vivo* and led to complete tumor clearance in mice bearing metastasized B16F10 melanoma. Further development led to stimuli-responsive particles that release IL-15 superagonist upon increased redox activity at the T cell surface upon TCR signaling (51). To enable repeated *in vivo* stimulation of adoptively transferred T cells, particles were designed that target T cells through an αThy1.1 antibody or IL-2 (50). To circumvent the need to culture cells *ex vivo*, αCD3 antibody fragments can be used to target biodegradable poly(β-amino ester)-based nanoparticles to T cells *in vivo*. These nanoparticles contained a DNA plasmid encoding a leukemia-specific CAR gene combined with 4-1BB and CD3ζ cytoplasmic signaling domains. This strategy resulted in the *in vivo* generation of CAR-T cells that perform comparable to CAR-T cells generated using the conventional *ex vivo* culture method (106).

Most biomaterials for T cell activation are designed to function in soluble form or in suspension as two-dimensional systems. Limited work has been performed using three-dimensional (3D) scaffold-based designs for T cell activation, whereas within the field of DC activation there are multiple examples of 3D scaffolds to create local DC-recruiting and activating niches. 3D scaffolds have proven advantageous as they present DCs with activating cues in a sustained manner at a localized site (107–109). We believe that designing such scaffolds and thereby creating synthetic immune niches for localized *in vivo* T cell activation could contribute significantly to the current T cell mediated anti-cancer therapies. A synthetic immune niche as a site of T cell priming and dispersion could replace or augment the function of tumor-draining lymph nodes, which were shown to be key regulators in the anti-tumor immune response (110).

One area in which 3D scaffolds have been explored for T cell activation is in the field of ACT (Figure 2C). An alginate implant equipped with T cell-stimulating signals (αCD3, αCD28, α4-1BB, and IL-15 superagonist) and migration-promoting peptides induced a substantial increase in the proliferation of adoptively transferred T cells at the tumor resection site in a 4T1 mouse breast tumor model (100). Moreover, these cells did not acquire an exhausted phenotype, but migrated toward the tumor-draining lymph nodes where they differentiated into central memory T cells. In another alginate-based scaffold approach, a combination of adoptive transfer of CAR-T cells and the incorporation of a STING agonist was used to trigger anti-tumor host immunity (53). A chitosan thermogel has also been tested for ACT and resulted in a supportive environment where antigen-specific T cells could proliferate and subsequently migrate toward target cells, which were effectively killed *in vitro* (111). Mesoporous silica micro-rods with supported lipid bilayers were used to provide T cells with either polyclonal cues (αCD3) or antigen-specific cues (pMHC) in combination with αCD28 for co-stimulation and adsorbed IL-2 to provide paracrine delivery of cytokines (54). Alternatively, a fully-synthetic hydrogel composed of tri-ethylene glycol-substituted polyisocyanopeptides functionalized with integrin-binding motifs supported the *ex vivo* expansion and survival of T cells (55). The *ex vivo*-stimulated T cells could successfully egress from the hydrogel over time when administered *in vivo*, identifying these hydrogels as effective cellular delivery vehicles. Together, these studies demonstrate proof of the concept that 3D scaffolds can be used as a multifunctional platform to enhance polyclonal and antigen-specific T cell expansion and cell persistence *in vivo*.

The use of biomaterials for the adoptive transfer of T cells might make ACT more efficient, as *ex vivo* culture time could be reduced and be potentially superfluous. This would make ACT more feasible and at the same time benefit T cell functionality (105). Moreover, implementing molecular cues like IL-7, IL-15, or IL-21 in 3D biomaterial-based scaffolds could provide an *in vivo* immune niche for the generation and support of adoptively transferred Tscm, which in turn could improve *in vivo* T cell persistence, proliferation and anti-tumor response (71, 78), underlining the promise of biomaterial-based 3D scaffolds for T cell activation *in vivo*. Careful investigation of the behavior of T cells in response to different combinations of molecular cues is required to create the most desirable T cell-activating synthetic immune niche.

## Concluding Remarks

Biomaterials are highly promising tools to present molecular cues to T cells to evoke robust immune responses both *in vitro* and *in vivo*. In this perspective, we presented an overview of molecular cues that could be used to selectively expand T cell subsets that are beneficial for strong anti-tumor immune responses. Biomaterials can be exploited to control the presentation of specific combinations of these molecular cues to T cells and can thus be used to regulate the stimulation level and the induction of specific T cell phenotypes. Careful consideration of how to combine the insights on important T cell-activating molecular cues with material-intrinsic factors is highly important for the design of biomaterials for the expansion and activation of tumor-specific T cells. In this respect, biomaterials could be used as tools to delineate the molecular cues and scaffold design parameters that dictate T cells' responses. When designing biomaterials for controlled activation of the immune system, it is important to take into account the intrinsic immunogenicity of materials and the potential change in immunomodulatory properties after biodegradation (112, 113). In our opinion, one of the major factors that needs to be considered is implementing potent CD4^+^ T helper cues alongside CD8^+^ T cell signals on biomaterials. In particular, tuning the T helper response toward a more Th1 and/or Th17 response might have considerable effects on clinical outcomes. Moreover, an improved understanding of the cues essential for memory cell formation is needed in order to develop biomaterial designs that can elicit long-term memory and thus better protect against tumor recurrence. It will be imperative to pursue a balanced and controlled system in terms of number and the release kinetics of molecular cues and the biodegradability of the biomaterial of choice (114).

As is evident from the studies discussed above, biomaterial-based cancer vaccines constitute a very promising field, but a number of challenges remain, especially those related to clinical translation. The ultimate goal is clinical application of biomaterial-based systems to induce long-term and systemic anti-cancer immunity in cancer patients. To ensure smooth transition to clinical translation, it is important to recognize key design parameters from the beginning of the design process, such as biomaterial composition, reproducible and large-scale production under good manufacturing practice (GMP), *in vivo* behavior, degradation, toxicities and safety. This can contribute to decreasing the time and cost of the regulatory pathway. Cell-free biomaterials for local administration, like 3D scaffolds, typically need less extensive testing to get approval, due to limited risks of systemic toxicities (115). However, local toxicity and inflammation may still arise and need to be carefully tested. The soluble biomaterial strategies, such as the particle-based aAPCs, might be considered as biologicals which would indicate a longer and more expensive regulatory pathway. Moreover, modifying already existing and approved therapeutic designs, including materials such as poly(lactic-co-glycolic acid) (PLGA) and hyaluronic acid, will allow for a clearer regulatory pathway (115). The field of regenerative medicine has already contributed a range of clinically-approved biomaterial products from which biomaterial-based cancer immunotherapies may benefit (115, 116). Besides meeting the safety criteria, the biomaterial-based cancer immunotherapies will need to demonstrate efficacy in appropriate preclinical animal tumor models when compared to current therapies (116).

The backpacking of adoptively transferred cells using particle-based biomaterials is considered to have great clinical promise (51, 52). In the beginning of this year, a phase 1 clinical trial with these biomaterial-based T cell backpacks started in patients with solid tumors and lymphomas (117). This example, together with the developments and future directions described in this perspective, illustrates that innovative designs of biomaterials for the direct activation of T cells will bring clinical implementation of biomaterial-based expansion and differentiation of tumor-reactive T cells closer.

## Author Contributions

MS, RH, CF, MV, and JW contributed to researching the data for the article, discussing the content and to reviewing and editing the manuscript before submission. MS, MV, and JW were responsible for writing the article.

### Conflict of Interest Statement

The authors declare that the research was conducted in the absence of any commercial or financial relationships that could be construed as a potential conflict of interest.
